# Mapping the social network: tracking lice in a wild primate (*Microcebus rufus*) population to infer social contacts and vector potential

**DOI:** 10.1186/1472-6785-12-4

**Published:** 2012-03-26

**Authors:** Sarah Zohdy, Addison D Kemp, Lance A Durden, Patricia C Wright, Jukka Jernvall

**Affiliations:** 1 Institute of Biotechnology, University of Helsinki, Viikinkaari 9, P.O. Box 56 Helsinki FIN 00014, Finland; 2 Department of Biology, Georgia Southern University, 69 Georgia Avenue, Statesboro, Georgia 30460-8042, USA; 3 Department of Anthropology, Stony Brook University, Circle Road Social & Behavioral Science Building Stony Brook, New York 11794-4364, USA; 4 Centre Val Bio, Ranomafana, Ifanadiana 312, Madagascar

**Keywords:** Primate, Parasite, Lice, Social contact, Mouse lemur, Vector potential

## Abstract

**Background:**

Studies of host-parasite interactions have the potential to provide insights into the ecology of both organisms involved. We monitored the movement of sucking lice (*Lemurpediculus verruculosus*), parasites that require direct host-host contact to be transferred, in their host population of wild mouse lemurs (*Microcebus rufus*). These lemurs live in the rainforests of Madagascar, are small (40 g), arboreal, nocturnal, solitary foraging primates for which data on population-wide interactions are difficult to obtain. We developed a simple, cost effective method exploiting the intimate relationship between louse and lemur, whereby individual lice were marked, without removal from their host, with an individualized code, and tracked throughout the lemur population. We then tested the hypotheses that 1) the frequency of louse transfers, and thus interactions, would decrease with increasing distance between paired individual lemurs; 2) due to host polygynandry, social interactions and hence louse transfers would increase during the onset of the breeding season; and 3) individual mouse lemurs would vary in their contributions to the spread of lice.

**Results:**

We show that louse transfers involved 43.75% of the studied lemur population, exclusively males. Louse transfers peaked during the breeding season, perhaps due to increased social interactions between lemurs. Although trap-based individual lemur ranging patterns are restricted, louse transfer rate does not correlate with the distance between lemur trapping locales, indicating wider host ranging behavior and a greater risk of rapid population-wide pathogen transmission than predicted by standard trapping data alone. Furthermore, relatively few lemur individuals contributed disproportionately to the rapid spread of lice throughout the population.

**Conclusions:**

Using a simple method, we were able to visualize exchanges of lice in a population of cryptic wild primates. This method not only provided insight into the previously unseen parasite movement between lemurs, but also allowed us to infer social interactions between them. As lice are known pathogen vectors, our method also allowed us to identify the lemurs most likely to facilitate louse-mediated epidemics. Our approach demonstrates the potential to uncover otherwise inaccessible parasite-host, and host social interaction data in any trappable species parasitized by sucking lice.

## Background

Ectoparasites have evolved with their hosts for millenia, resulting in highly intimate host-parasite relationships [1,2]. Due to the intimacy of such relationships, studies of host-parasite interactions have the potential to provide novel insights into the ecology of both organisms involved and data collected on one of the partners can have implications for the other. Previous studies have directly monitored host resource usage and contacts to provide improved predictions about parasite and pathogen transmission [3]. However, direct observation of social interactions is not feasible in species that are nocturnal, arboreal, subterranean or otherwise elusive. Additionally, direct transfers of ectoparasites between individually identifiable hosts has been difficult to document. In this study we employ a novel method of tracking lice to collect empirical data on host social interactions, as well as reveal parasite-host interactions.

The hosts studied were brown mouse lemurs (*Microcebus rufus*) of southestern Madagascar's tropical montane rainforests which, at 40 g, are one of the world's smallest primates. They are arboreal, noctural and cryptic, which has impeded collection of data on their social interactions despite advances in relevant technology. They are the sole known host of *Lemurpediculus verruculosus *[4], one of the ~540 described species of obligate, permanent blood-feeding sucking lice (Anoplura) that parasitize 12 of the 29 mammalian orders [5]. Sucking lice have evolved a morphology that is highly specialized for life on their hosts, including features that permit secure attachment to host hair and feeding directly upon host peripheral vasculature. This specialization confers considerable advantages to sucking lice while they are on the host; however, it also restricts the amount of time lice can spend off a host to just a few hours [6]. This limitation necessitates that louse transfers between hosts occur when two or more host individuals are in direct contact. Though some chewing lice are known to transfer between hosts phoretically by attaching to winged hippoboscid flies [7] and other parasites can transfer fomitically via inanimate objects [8], the highly specialized features of sucking lice preclude the use of these transfer routes [6].

Understanding patterns of sucking louse transfer is particularly important because some species are known to transmit blood-borne pathogens during their bloodmeals. Sucking lice parasitizing domestic animals are known to transmit pathogens such as poxvirus (pigs), *Mycoplasma *(formerly *Eperythrozoon *and *Haemobartonella*) (rats and mice) [9]*Anaplasma *(goats, cattle, pigs), and *Rickettsia *(goats, cattle) [10], while human sucking lice transmit the pathogens responsible for trench fever (caused by *Bartonella quintana*), epidemic typhus (*Rickettsia prowazekii*) and epidemic relapsing fever (*Borrelia recurrentis*) [11]. Despite the well-established relationship between sucking louse transfer and the spread of louse-borne disease agents in humans, very little is known about sucking louse transfer in wild mammals. Thus, documenting louse transfer permits assessment of host vulnerability to vector-borne pathogens.

Live-trapping data on the population of *M. rufus *at Ranomafana National Park indicate that individually identifiable lemurs are highly localized to their mode trap locale and are infrequently found in trap locales that would imply wide ranging. This implies that mouse lemurs would be most likely to come into contact primarily with their nearest individuals. Based on this, we hypothesized that the frequency of louse transfers, and thus interactions, would decrease with increasing distance between the trap locations of paired individuals. Because *M. rufus *are solitary foragers with a polygynandrous mating system [12] their brief annual breeding period in early October [13] necessitates an increase in social interactions; we therefore hypothesized there would be an increase in louse transfers during the onset of the breeding season. Given the limited apparent ranges of the lemurs and the variation in number of near neighbours an individual may have, we also predicted that individual hosts would vary in their contributions to the overall parasite ecology of the population. Sucking lice are one of the most host dependent groups of ectoparasites and from their perspective the parasite-host relationship is completely inextricable.

Based on the unique, direct link between sucking louse transmission and host contact, we developed a method of marking lice (without their removal) with an individualized dot code and monitoring their movement through the host population to test hypotheses regarding host ranging patterns, social interactions, and individually varying roles in overall population parasite ecology.

## Results

All lice recorded were found on the ears, testes and eyelids (see Additional file 1: Figure S1a for an image of the lice observed on the testes). We observed all stages of the *L. verruculosus *life cycle on the testes (see Additional file 1: Figure S1b for an image). We found no evidence that capture rate was related to mean louse intensity or the percentage of marked lice a host would eventually transfer, with only 19% of variation in the former and 21% in the latter being explained by these variables.

**Figure 1:**
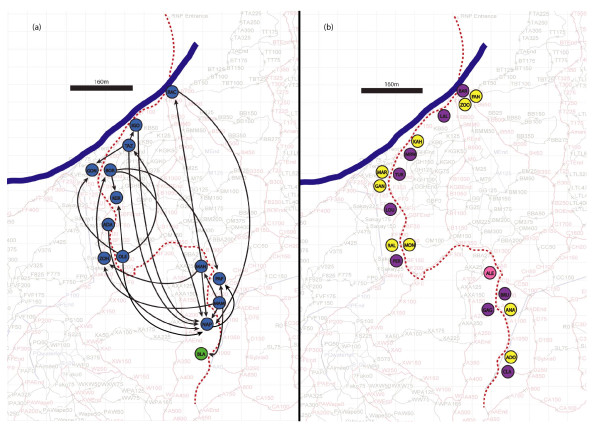
**Map of lemur social contacts, spatial plots of lemur trapping locations and recorded louse transfers**. The dotted line represents the trapping transect (1km), the blue line represents the Namorona River, the circles represent the average trap locale for each lemur (identified with a three-letter code). **1a**. Schematic representation of recorded louse transfers. Blue circles are males that donated or donated/received; the green circle is the one male that only received lice. A black line connecting two dots indicates at least one recorded transfer between those lemurs. **1b**. Trap locales for lemurs which did not engage in recorded transfers. Yellow circles denote males not involved in observed transfers, purple circles denote females that never had lice, and pink circle denotes the only female that had lice.

Twenty-three male lemurs were captured; 13 individuals were infested with lice and had their lice marked, 9 never had lice, and an additional male never had unique lice but received a louse through transfer (Figure 1a). Nine females were captured; one was infested with lice but never engaged in transfer (Figure 1b). A total of 105 lice were marked and 76 transfers were recorded involving a minimum of 45 separate transfer events within a group of 14 male lemurs dispersed throughout the entire trapping transect (Figure 1a).

**Figure 2:**
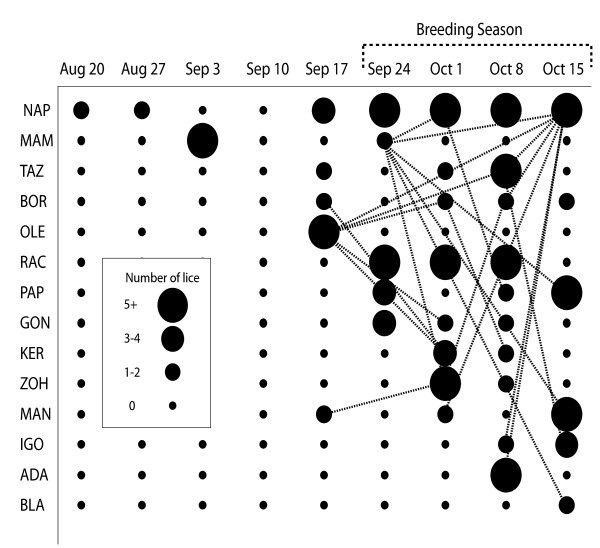
**Pre-breeding and breeding season louse infestation intensities and transfers in the 14 male lemurs involved in transfers**. Bubble sizes correspond to the combined number of new lice on a lemur as well as previously marked lice recaptured on it. Data for multiple trapping dates have been combined into weeks for simplification. The bracketed area represents the breeding season, with peak breeding occurring in early October. Lines represent louse transfer events, during which one or more lice transferred. Transfer events are always indicated as beginning on the host. Thus, if an individual's louse/lice engaged in multiple transfers (first to a second lemur, then via the second lemur to a third) there will be two lines from the original host on the day the louse was marked - one to the second lemur in the week the louse was recaptured, and one to the third lemur on a different date. Louse intensities began increasing in the population and transfers began occurring two weeks before the peak breeding season.

Louse intensities were low (mean < 1 per lemur per week) for the first four weeks of trapping, and began to increase the week before the breeding period. This was also the week from which the first set of recorded transfers originated and the first week that lice were found on the testes. The majority of the marked recovered lice (80.3%) were found on the testes of new hosts. Transfer rates and mean intensities remained high through the breeding season. Transfers increased in a stepwise manner at the onset of the breeding season rather than gradually with the total number marked lice. The percentage of marked lice that transferred remained zero until the beginning of the breeding season at which point 12% transferred despite a very slight previous increase (4%) in the marked louse population, suggesting that louse transfers are not a simple function of louse prevalence. One host individual (Ole) was infested with lice only once, just before the breeding season began, and had transferred all of his lice within a week; however, his lice continued to reappear in the population through the week after the breeding season (Figure 2). Similar evidence of lice from a single host engaging in multiple transfers was recorded for a second individual (Mam) beginning in the breeding season, one week before peak breeding. The individual with the highest overall louse intensity (Nap) (Figure 2) not only began the breeding season with a high number of his own lice, but was also the recipient of the greatest number of transferred lice.

**Figure 3:**
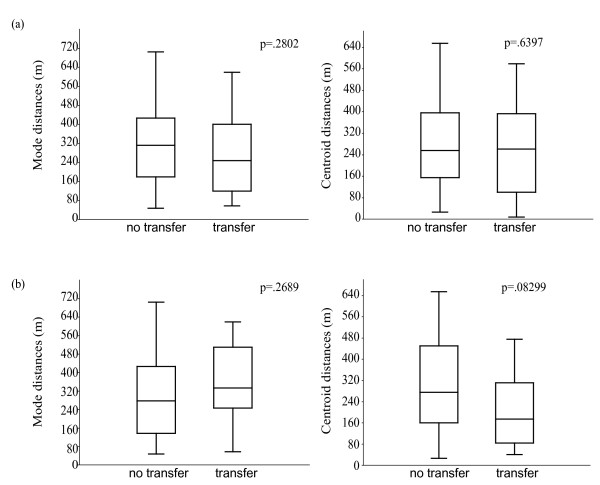
**Louse transfers are not limited to neighbouring lemurs**. **3a**. Louse transfers among all individual lemurs (**3a**) and excluding the four furthest ranging lemurs (**3b**) show that lemur-lemur distances measured using mode distance (distance between the most frequent capture locations) and centroid distances (weighted centroid of all the capture locations of each lemur) show non-significant differences between those lemurs that participated and those lemurs that did not participate in louse transfers. Note that centroid distances of lemurs excluding four furthest ranging individuals suggest that louse transfers are more frequent between neighbours (**3b**). Boxes enclose 50% of observations; the median is indicated with a horizontal bar, and whiskers denote range.

For all 14 males involved in louse transfers, each individual's mode trap location was used to calculate pair-wise distances. Pair-wise distance calculations were also made based on the centroid of each individual's trap locales. The absolute minimum distance a transferred louse was observed to travel on its hosts based on mode trap locale was 57 m and the maximum distance was 634 m. Centroid-based calculations yielded a minimum louse transfer distance of 7.4 m and a maximum of 578 m. We found no significant difference in pair-wise distances calculated from mode or centroid values between the pairs of animals that transferred lice and those that did not (Figure 3a) (p = 0.28 for mode distances and p = 0.64 for centroid distances), indicating that the probability of animals transferring lice across long distances and short distances is comparable. Therefore, rather than supporting our hypothesis that lice are most frequently exchanged between neighbouring lemurs, the data indicate that louse transfers occur across short and long distances.

Plotting the number of times an animal was captured against that animal's total number of trap locales showed that most animals were trapped at up to 4 trap locales (see Additional file 2: Figure S2a for this plot). However, there were four individuals that were captured more than 11 times at 5 or more trap locales which also showed the longest maximum distance between trapping locales (see Additional file 2: Figure S2b). We reran the louse transfer analysis removing those four individuals and the results show that the centroid distances between lemur transferring lice were now shorter compared to lemurs that did not transfer lice, although this difference remained non-significant (Figure 3b).

Social network analysis exposed that only 8 of the 28 predicted social contacts based on trapping data also were involved in louse transfers; however, 13 unique social contacts based on louse transfers alone could not be accounted for based on trapping data. Therefore, there is evidence that the louse marking technique revealed a lemur social network that would not have been derived using trapping data alone (see Additional file 3: Figure S3, Additional file 4: Figure S4, and Additional file 5: Table S1).

We recorded at least three lice that were each involved in multiple recorded transfers, beginning on one lemur, then being recaptured on at least two more. These multiple transfers were identified conservatively and only when that was the sole possible explanation for the distribution of lice from a given host. The longest period a single louse remained attached to its host was 30 days. The locations in which lemurs were trapped did not cluster based on lemur sex or involvement in louse transferal. Transfers occurred over a range of distances up to the full length of the transect and occurred evenly throughout the trapped area.

Donor scores (see Methods) ranged from +10 to -18 and the vector potential scores (see Methods) varied considerably throughout the population. This variation was not significantly related to the variation seen in donor scores (R^2 ^= 0.47, p = 0.69), but the lemur with the highest donor score also had the greatest vector potential (Table 1).

**Table 1:** Summary of factors influencing the overall role of individual male mouse lemurs in louse transfers within the lemur population and the potential for that host to transmit a vector-borne pathogen via louse transferal

Lemur	Mean body mass (g)	Age (yrs)	Testicular volume (mm3)	Total lice marked	Number of times captured	Number of lice donated	Number of lice received	Number of hosts donated to	Vector potential	Donor score
Mam*	51.00	5.38	526.33	9.00	11.00	9.00	0.00	5.00	*5.00*	*10.00*

Ole*	43.00	2.72	321.58	7.00	3.00	7.00	0.00	4.00	*4.00*	*8.00*

**Ada**	56.00	1.06	678.40	5.00	1.00	5.00	0.00	1.00	*1.00*	*5.00*

Man*	42.00	1.97	280.46	5.00	6.00	5.00	2.00	3.00	*3.00*	*5.00*

**Bor**	52.00	3.49	584.01	4.00	8.00	2.00	1.00	3.00	*1.50*	*1.00*

**Igo**	49.00	1.48	407.09	2.00	9.00	2.00	1.00	1.00	*1.00*	*1.00*

**Zoh**	54.00	3.03	704.88	4.00	3.00	1.00	3.00	1.00	*0.33*	*0.00*

**Rac**	49.60	0.72	619.02	34.00	10.00	1.00	1.00	1.00	*0.03*	*0.00*

**Bla**	42.00	2.84	447.10	0.00	4.00	0.00	1.00	0.00	*0.00*	*-1.00*

**Gon**	38.30	1.42	274.05	5.00	12.00	0.00	1.00	0.00	*0.00*	*-1.00*

**Taz**	47.25	3.90	476.20	2.00	9.00	2.00	3.00	2.00	*2.00*	*-1.00*

**Ker**	49.00	4.42	522.69	2.00	11.00	0.00	2.00	0.00	*0.00*	*-2.00*

**Pap**	45.75	1.60	512.85	3.00	12.00	0.00	5.00	0.00	*0.00*	*-5.00*

**Nap**	47.10	.21	549.76	22.00	14.00	2.00	20.00	1.00	*0.09*	*-18.00*

Individuals with an asterisk * are those whose individual lice transferred multiple times to different hosts. These multiple transfers also resulted in the discrepancy between the totals of 'received' and 'donated' lice.

Of the 14 mouse lemurs involved in louse transfers, those individuals that donated 50% or more of their marked lice received on average 1 ± 1.15 lice from other individuals. Lemurs that donated less than 50% of their marked lice received on average 4.3 ± 7.1 lice (Additional file 6: Figure S5 and Additional file 7: Table S2). When examining the number of lice donated and received out of the total number of lice (not just the marked ear lice) we find similar results. Lemurs that donated 50% or more of their total lice received fewer lice (on average 0.6 ± 0.89) than those that donated less than 50% of their total lice and received on average 7.0 ± 14.69 lice. This distribution suggests that while mouse lemurs may fall along a continuous range of values, there is evidence of two louse transfer categories: 'donors' that are less likely to receive and more likely to donate lice, and 'recipients' that are more likely to receive and less likely to donate lice.

## Discussion

We were able to successfully track patterns of louse movement in a population of wild *M. rufus*. Recorded transfers occurred exclusively between males which may have been due to male-male contact resulting from nest-hole sharing [14], agonistic interactions occurring during the breeding season [15], or from multiple males mating with the same female. No females, however, were ever found to have lice on or near the genitals. The transfers were quite evenly distributed throughout the study area and showed no consistent distance, direction or clustering patterns. This pattern of transfer contrasts with mouse lemur trapping data in the study area. Most of the louse transfers occurred between lemurs over 100 m from each other, and one transfer (between Rac and Nap, Figure 1a) spanned over 600 m. The transfers therefore demonstrate a degree of lemur ranging far greater than anticipated using standard trapping methods (Additional file 8: Table S3). They also provide evidence of a lemur social network that could not have been predicted based on trapping data alone (Additional file 3: Figure S3, Additional file 4: Figure S4, and Additional file 5: Table S1). Moreover, relatively few individual lemurs with broad ranging patterns appear to be largely responsible for the long-distance transfer of lice. These patterns of parasite-host dynamics suggest that a small number of individuals can quickly spread parasites throughout the population.

As hypothesized, transfers increased significantly at the beginning of the breeding season, indicating an increase in host interactions. We found that transfers occurred most frequently and nearly exclusively during the breeding season. This combined with the restriction of transfers to male hosts suggest that the transfers were due to agonistic same-sex interactions associated with the breeding season. A previous study [16] in chipmunks indicated that breeding periods correspond to an increase in rates of louse transfer, but there are also arguments that louse transfer in general is not a common occurrence [6,16]. The 76 transfers we tracked between 14 animals over the course of four weeks are therefore remarkable both with respect to the high rate at which they occurred, and their correlation with the breeding season.

Although no marked lice were recaptured on female lemurs, it is possible that they were involved as intermediaries in male-male transfers. All transfers observed required at least one incidence of direct contact; however, it is possible that an intermediate host could have facilitated these transfers between what we record as the donating and receiving hosts. Thus it is possible that a female received a louse from one male during a copulation event and transferred it to second male during a later copulation. This is unlikely to be a common occurrence, however, as we only observed a single louse on a single female host over the entire duration of the study. Additionally, testosterone is known to increase the transmission potential of certain parasites [17], suggesting that the high testosterone levels seen in male mouse lemurs may increase the transmission potential of louse-borne pathogens. Testosterone has also been implicated as an immunosuppressant [18-22], and during the reproductive season animals experiencing increases in testosterone are more likely to be vulnerable to parasitic infestation [17,23,24]. An increase in testosterone levels as the males invest in spermatogenesis for the annual breeding season may be a mechanism underlying the all-male transfers.

Lice previously marked on the ears of one host were most frequently recaptured on the testes of that same host or other hosts, rather than the ears. The frequent migration of lice to the testes and the presence of all stages of the louse life cycle there indicate that this is a preferred attachment site, potentially because of the area's rich peripheral blood supply and relatively sparse fur leading up to and during the breeding period when they are dramatically distended (see Additional file 9: Figure S6 for an example). The timing of this increase in louse populations about one week before host breeding indicates that lice may be triggered to reproduce by increasing levels of host sex hormones in their bloodmeals as occurs in at least two species of fleas that parasitize rabbits [25]. This could explain the appearance of lice on the testes immediately before the beginning of the breeding season. We found no correlation between louse intensity and testicular volume in the 14 males involved in transfers (Table 1, also see Additional file 10: Text S1 for more information).

The variation in donor scores indicates that host individuals do play varying roles in the transfer of lice and transmission of their potential pathogens throughout the population. Age of the host may be a factor determining which hosts act more as donors. Durden (1983) [16] observed that juvenile hosts did not act as donors in louse transfers despite having larger louse infestations than adults. Indeed, the youngest host in this study had the heaviest louse infestation but donated only one louse. However, we found no overall correlation between the age of the host and louse transfers (Table 1, also see Additional file 10: Text S1 for more information), and the divide between 'donors' and 'recipients' does not appear to be the result of testicular volume, age or body mass.

Vector potential scores, like donor scores, ranged widely between individuals; however, the two are not significantly correlated. Vector potential scores therefore provide a second distinct way of assessing the impact an individual lemur host can have on patterns of louse transmission and population-wide pathogen transmission. The evidence of multiple host transfers also suggests a heightened probability of potential pathogen transmission as the probability for lice contacting and carrying a blood-borne pathogen increases with exposure to blood from an increasing number of hosts. Of the four animals removed from the distance analysis (Figure 3) one individual (Mam) was responsible for transfers over both short and long centroid-based distances. Interestingly, this same male was revealed by the vector potential analysis (not influenced by distance variable) to be the starting point for the greatest number of transfers.

Lice act as vectors of blood-borne pathogens in other host species and two blood-borne pathogens found in lemurs, *Babesia cheirogalei *and *Babesia propitheci*, are known to be transmitted by ectoparasites (especially ticks) [26]. It remains to be determined if *L. verruculosus *transmits blood-borne pathogens. *Microcebus *spp. are found throughout the island of Madagascar and often come into close contact with humans through a combination of anthropogenic habitat destruction and their generalist behavior; thus it is potentially beneficial to understand their parasite ecology and its implications not only for their population but for other species, including humans, as well.

Beldomenico and Begon [27] proposed that animals with poor body conditions are more susceptible to parasitic/pathogenic infections, which in turn would perpetuate their poor body conditions. They refer to this as a 'vicious circle' which results in heavily infested animals that become 'superspreaders', who disproportionately contribute to the dispersal of parasites in a population. Our technique of following the movement of lice through a wild population revealed the presence of lemurs that did disproportionately contribute lice to the rest of the population, making them 'donors' or 'superspreaders'. However, the mouse lemurs most heavily parasitized in this study were the ones that collected the most lice from others and hence not superspreaders, but rather 'recipients' or 'supercollectors'. Heavy louse infestation may be due to poorer overall body condition; however, as is seen in Table 1 (and Additional file 10: Text S1) we found no significant trend with body mass, age or testicular volume that would suggest that the supercollectors have the poorest body conditions.

The superspreaders suggested by [27] as the primary carriers and distributors of parasites may make parasite populations vulnerable, as the host's poor body condition would result in an increased likelihood of being preyed upon [28-30]. We suggest that rather than superspreaders simultaneously occurring as heavily infested supercollectors, the presence of both types of individuals occur independently in wild populations. Perhaps with further research tracking parasites in natural habitats, alternate disease dispersal routes will be revealed.

## Conclusions

In conclusion, this study capitalizes on the biological features of lice to gather otherwise inaccessible social interaction and population parasite ecology data on its host, *M. rufus*. We found indications of far greater lemur ranging distances and a more widespread social network than were estimated using live-trapping data. We also found that although there is no evidence of louse transfer before the breeding season, there is a substantial increase in transfer rate as the season begins, implying an underlying acceleration in rates of direct social contact. This increase in transfer rate continues through the host breeding season, as the rate of social interactions presumably increases. Finally, we determined that while different lemur individuals play varying roles as louse donors and recipients, all individuals would be at far greater risk from an introduced louse-borne pathogen during the breeding season due to the high rate at which it could spread through the male population. The approach developed here has potential for application in any species parasitized by sucking lice, including the many trappable species of cryptic, nocturnal, subterraneous or otherwise elusive mammals in which host social contact and parasite exchange data are difficult to obtain.

## Methods

### Study site and host species

Research was conducted in Ranomafana National Park (RNP) (47° 18' - 47° 37' E, 21° 02' - 21° 25' S), a montane rainforest in southeastern Madagascar. RNP includes 43,500 hectares of continuous rain forest from lowland to montane habitats receiving a mean of 3000 mm of rain a year [31,32]. This forested area was selectively logged in the 1980s, and is now visited by tourists [33]. A transect along the Talatakely Trail system was used for *M. rufus *trapping from August 1 through October 31, 2010, a period encompassing their annual breeding season. Mouse lemurs are seasonal breeders with a single brief period per year during which they can mate. The start of the breeding season was determined based on the estrous state of females of the population, using vaginal morphology as an indicator. We observed a two-week breeding period in early October, during which females are receptive to copulation (open) for only a few days. This pattern matches data from previous studies on *M. rufus *reproduction [12,13].

Field research was completed under research permits #115/10 MEF/SG/DGF/DCB.SAP/SCBSE, and #215/08 MEFT/SG/DGEF/DSAP/SSE Project ID#2009-1608. The use of animals was approved by the animal ethics committee of the University of Helsinki.

### Trapping

Thirty Sherman traps (XLR, Sherman Traps Inc., Tallahassee, FL) were set in trees at standardized paired sites placed along selected trails at intervals of 20-50 meters. To maximize trapping effort, traps were set on every other night during the study period. Traps were baited with fresh banana and set at 16:00; they were then checked a few hours later, between 19:30 and 20:00. Non-primate captures were identified, noted and released. Captured mouse lemurs were handled by a trained research technician, individually scanned for a microchip (AVID Powertracker VI), sexed, weighed and given a microchip if needed. Testicular length and width (mm) were measured in males and volume was calculated using the formula for an ellipsoid V = (π × TL left × TW^2 ^left)/6+ (π × TL right × TW^2 ^right)/6 [34]. Individuals were aged using the methods described in [35]. All mouse lemurs of both sexes were equally examined for lice. Using a flea comb, the individuals were searched for lice beginning from the back of the head, down the left dorsum, then the right dorsum including both arms. Then the ventrum was examined beginning with the ears, down the face, then down the left side of the body, followed by the right side of the body. Due to the large size of the lice (approx 1 mm) relative to the length of the mouse lemur from head to base of tail (approx 100 mm) the lice were quite easily visible on the lemurs. The amount of time it took to examine a lemur varied depending on the lemur. Some lemurs are quite active while being handled (and hence take longer to process), while others remain still (and hence take less time to process). However, the overall body examination rarely exceeded 10 minutes. Following louse marking, mouse lemurs were released at the site where they were captured.

### Louse marking

**Figure 4:**
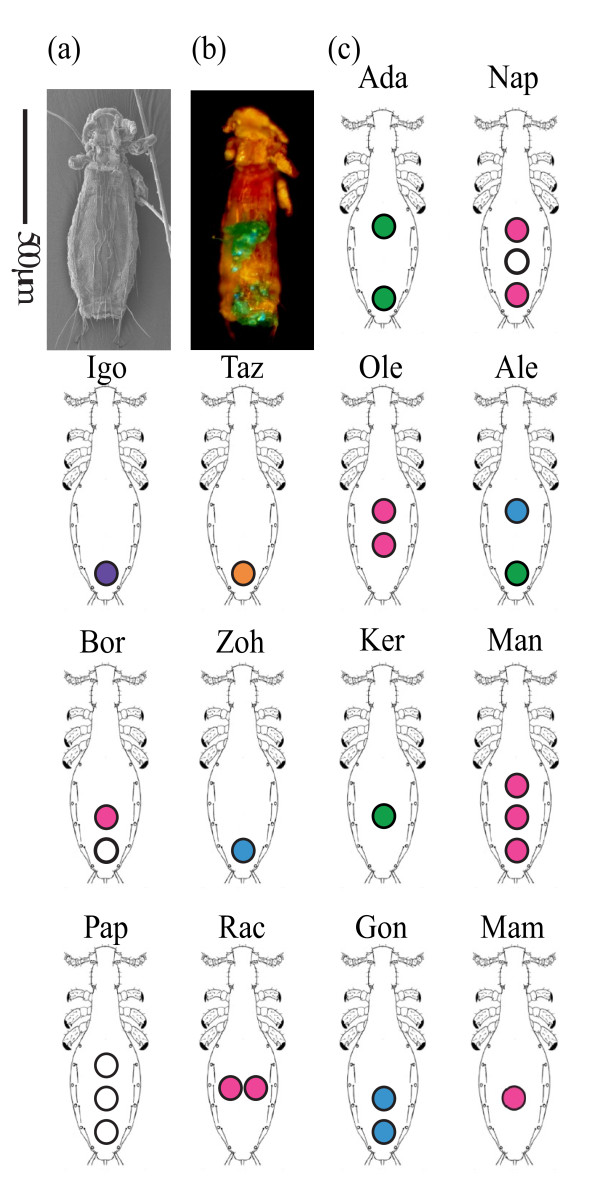
**Marking system for *L. verruculosus***. **4a**. Scanning electron micrograph (SEM) of a *L. verruculosus *female dorsal view. **4b**. Photograph of a female louse marked using the techniques in this study. Separate green markings on the anterior and posterior dorsal abdomen represent host identification "Ada". The red portion is blood visible from louse feeding. **4c**. Schematic drawings of the individualized codes assigned to lice. The three letters above each schematic are the host codes that refer to individual mouse lemurs. Scale bar is 500 μm. Louse drawing modified from Durden et al. (2010) [10] with copyright permission from Allen Press.

Each mouse lemur was assigned a unique code for its lice consisting of up to three colored dots placed in varying patterns between anterior, mid, and posterior dorsal abdomen (Figure 4). These host codes were placed on all of the lice found on the ears of the lemurs using a linen test magnifier with LED light (Clas Ohlson, Insjön, Sweden), sharpened toothpick applicator, and several different colors of nail lacquer (Hennes & Mauritz AB, Stockholm, Sweden). To minimize the amount of time that the lemurs were being handled, we only marked the lice on the ears, a location sparse in hair in which the lice are easily visible. The ears are also the area of the body that we previously found to be the most heavily parasitized [4]. All lice found on mouse lemur ears were marked without removal. Markings were given 20 seconds to dry. Host codes were selected such that wear or removal of a dot would not cause misidentification and the texture of the louse surface integument makes it highly unlikely that a marking would fall off. Further, throughout the duration of the study no recovered louse ever had a dot code that could not be accounted for. Lice were marked as they were found on hosts throughout the entire duration of the study. When the hosts were trapped, all lice, including those previously marked, were recorded and their position on the host body was noted.

### Analysis

The mode trap locale of all host individuals involved in transfers was documented on a map of the trapping site and the 91 pair-wise distances (in meters) were calculated to create a distance matrix of interactions. In addition, the centroid locale for all individuals involved in transfers was calculated to gain a better estimate of each individual's home area based on trapping locations. This was calculated based on the number of times an individual was captured at different trap sites and weighted accordingly. The 91 pair-wise distances were then calculated using trap locale centroids for comparison. A Mann-Whitney *U *test was conducted to test for significant differences between pair-wise distances of those pairs that did and did not transfer lice. This was done for both distances between centroids and distances between mode trap locales. Additionally, the four individuals that were captured the most times at the greatest number of unique trap locales were removed and the analysis was rerun with similar results.

To address whether or not the social network derived from louse marking data was the same as a network predicted based on trapping data alone, social network analysis was conducted using the social network analysis application in the program Gephi (Gephi consortium, Paris, France). Using this software, social contacts were derived based on overlapping trap locales; these were then compared to the contacts based on observed louse exchanges. These results were then mapped into a small world network and compared. Eigenvector centrality scores were then calculated to address whether the lemurs with the highest levels of connectedness were the same based on trapping data and louse marking data.

### Vector potential calculation

A donor score was calculated to determine whether individuals engaged in transfers acted predominantly as donors or receivers (Table 1). This score was calculated by subtracting the total number of lice received from the total number of lice donated by an individual. A vector potential score was calculated to assess the potential efficacy of individual lemurs as donors of vector (louse) transmitted pathogens. This score takes into account both the likelihood that a louse marked on an individual lemur will transfer, as well as the number of other hosts to which that lemur donated lice; thus it gives a rough measure of how widespread an impact a given lemur would have on the population if it contracted a louse-borne pathogen. The score was calculated for each individual as follows:

(number of lice donated×number of hosts donated to)/total numberoflicemarked

## Competing interests

The authors declare that they have no competing interests.

## Authors' contributions

SZ, LD designed the study. SZ and AK conducted the experiments. SZ, AK, JJ conducted the data analysis. SZ, AK, LD, PC, and JJ wrote the manuscript. All authors read and approved the final manuscript.

## Supplementary Material

Additional file 1 **Figure S1**. Infestation of *L. verruculosus *on the testes of a wild brown mouse lemur. 1a. Immediately preceding the breeding season, lice began to appear on the testes. 1b. All stages of the *L. verruculosus *life cycle are observed on the testes. All three nymphal instars and both sexes of the adult stage can be seen. Lemur testes had the greatest louse intensities (> 100 in some cases). Click here for file

Additional file 2 **Figure S2**. Frequency of capture suggests stereotyped trap locales for individual lemurs. 2a. Plotting the number of times an animal was captured (x-axis) against the total number of different trap locales in which an individual was caught shows that most animals were trapped at up to 4 trap locales. However, there were four individuals (in the ellipse) that were captured more than 11 times at 5 or more trap locales. 2b. These are the same individuals (in the ellipse) which also showed the longest maximum distance between trapping locales and appear to be largely responsible for the long-distance louse transfers recorded. Click here for file

Additional file 3 **Figure S3**. Social network map of mouse lemurs based on trapping data and louse exchanges. This figure represents the social contacts based on trapping data alone (black dashed line), based on louse transfers (dotted blue line), and contacts that occurred based on both trapping data and louse exchange data (solid purple line). 28 contacts were predicted based on trap locales, and 21 contacts were seen according to louse transfer data. Of the 21 louse exchange contacts, 8 of those were also paired based on trapping data; however, 13 lemur contacts based on louse exchanges can not be explained by trapping data. Of the 8 pairs with overlapping trap and louse contacts, 5 of those pairs belong to one individual (Mam), the same individual found to range widely throughout the trapping transect. These data suggests that while some lemur-lemur contacts may be predicted by trapping data, a majority of the louse exchanges seen in this study could not have been predicted based on trapping data alone. Additionally, the lemurs with the highest eigenvector centrality scores (indicating how well a lemur is connected to other lemurs) differed when calculating networks based on trapping and louse marking data separately. This means that calculating a social network based on trapping data alone would not have exposed the lemur with the most social contacts (Nap) as was revealed using louse marking data. Click here for file

Additional file 4 **Figure S4**. Individuals with a larger number of social connections calculated using trapping data also have a larger number of these same connections based on louse transfers (left, rs = 0.614, p = 0.027). In addition, louse transfer based calculations reveal additional contacts that could not have been predicted based on trapping data, and hence the number of contacts do not correlate significantly with trapping data based connections (right, rs = -0.417, p = 0.132). The three dot sizes represent one, two, and three individuals. Click here for file

Additional file 5 **Table S1**. Table showing eigenvector centralities calculated using social network analysis software. Click here for file

Additional file 6 **Figure S5**. Histogram of percentage of marked and total lice donated. In this figure the dark grey bars represent the percentage of lice donated out of the total number of lice found on the body. The light grey bars represent the percentage of lice donated out of the total number of marked lice from that individual. When including the total number of lice on the body (both marked and unmarked), five individuals donated more than 50% of their lice, and received on average 0.6 lice. The remaining individuals who donated less than 50% of their total lice received on average 7 lice. This suggests that whether examining the total proportion of lice donated, or the proportion of marked lice that were donated, the trends are the same, and individuals with more lice typically receive more lice from others, and individuals with fewer lice typically donate a larger percentage of them. There is no significant correlation between the total number of lice and the number of lice donated (r = -0.08, p = 0.079), or between the number of lice marked and the number of lice donated (r = 0.036, p = 0.90). Click here for file

Additional file 7 **Table S2**. Table showing the number of donated lice out of the total number of marked lice, and the total number of lice found on the body. Click here for file

Additional file 8 **Table S3**. Table representing the maximum distances lemurs travelled according to trapping data along with the maximum distances lemurs travelled according to the movement of their lice. Click here for file

Additional file 9 **Figure S6**. Testes of a brown mouse lemur during the breeding season. This image demonstrates the seasonal testicular growth seen in male mouse lemurs during the breeding season. The dotted line encircles the testicles. Click here for file

Additional file 10 **Text S1**. Additional text. Click here for file
