# Supplementary figures and images for: Mapping the social network: tracking lice in a wild primate (Microcebus rufus) population to infer social contacts and vector potential
Source: BMC Ecol. 2012 Mar 26;12:4. doi: 10.1186/1472-6785-12-4 (PMC3338373; doi:10.1186/1472-6785-12-4)

Supplemental Figure A1.

(a)

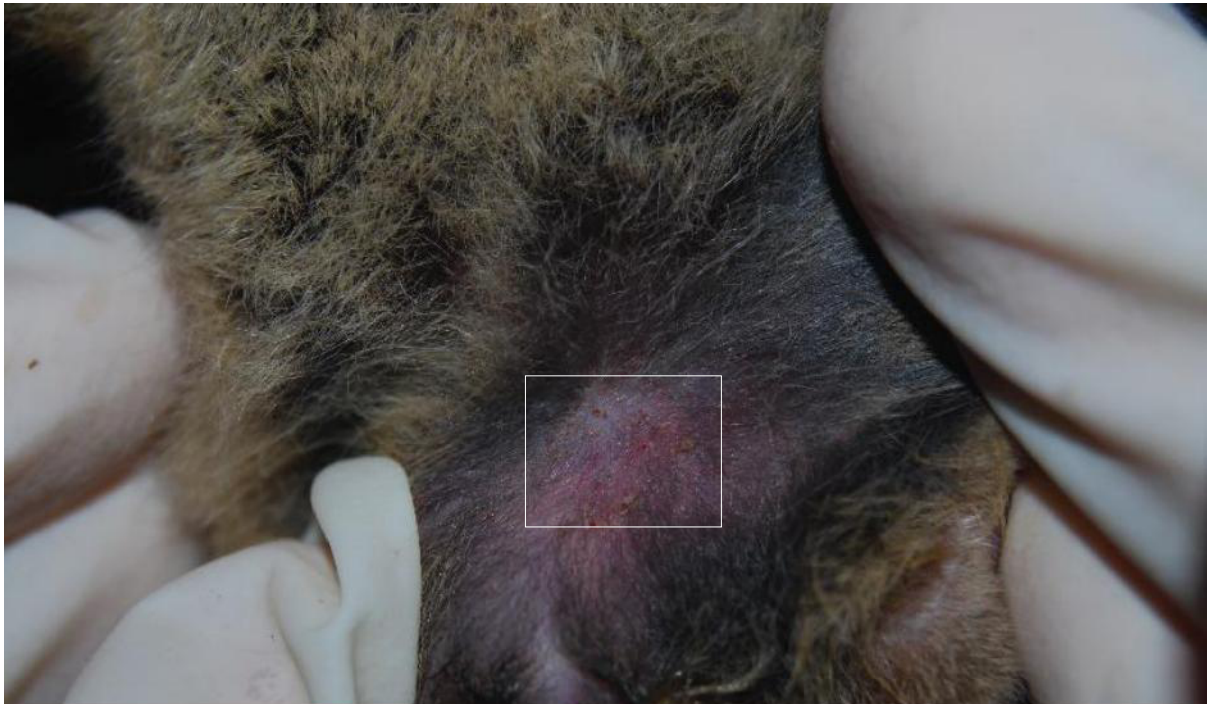

(b)

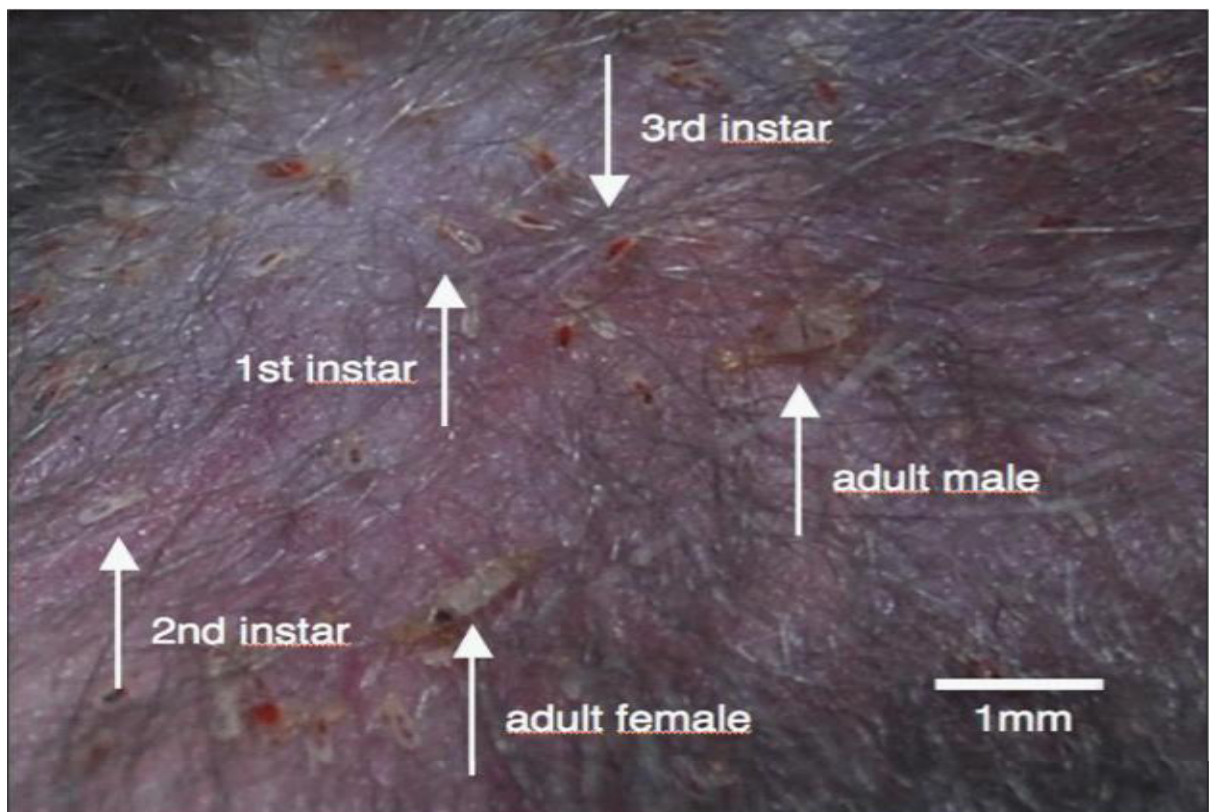

Supplement: Additional file 1 — Figure S1. Infestation of L. verruculosus on the testes of a wild brown mouse lemur. 1a. Immediately preceding the breeding season, lice began to appear on the testes. 1b. All stages of the L. verruculosus life cycle are observed on the testes. All three nymphal instars and both sexes of the adult stage can be seen. Lemur testes had the greatest louse intensities (> 100 in some cases). [file 1472-6785-12-4-S1.PDF]

(a)

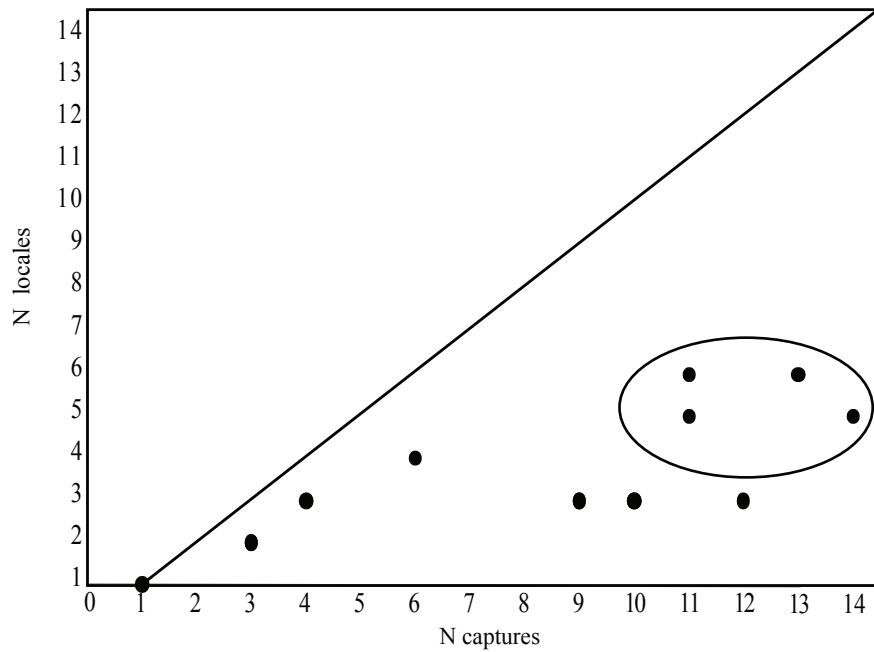

(b)

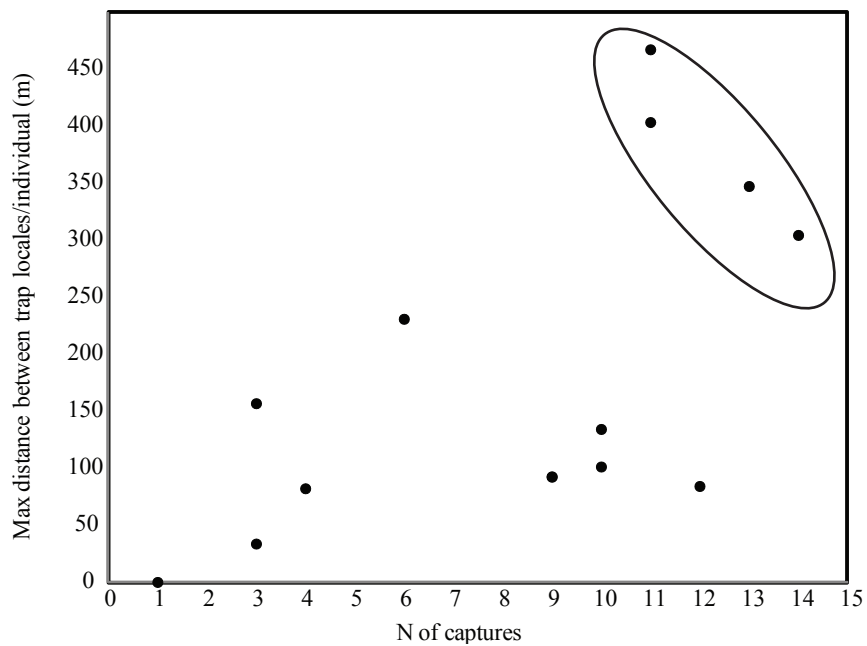

Supplement: Additional file 2 — Figure S2. Frequency of capture suggests stereotyped trap locales for individual lemurs. 2a. Plotting the number of times an animal was captured (x-axis) against the total number of different trap locales in which an individual was caught shows that most animals were trapped at up to 4 trap locales. However, there were four individuals (in the ellipse) that were captured more than 11 times at 5 or more trap locales. 2b. These are the same individuals (in the ellipse) which also showed the longest maximum distance between trapping locales and appear to be largely responsible for the long-distance louse transfers recorded. [file 1472-6785-12-4-S2.PDF]

Figure A3.

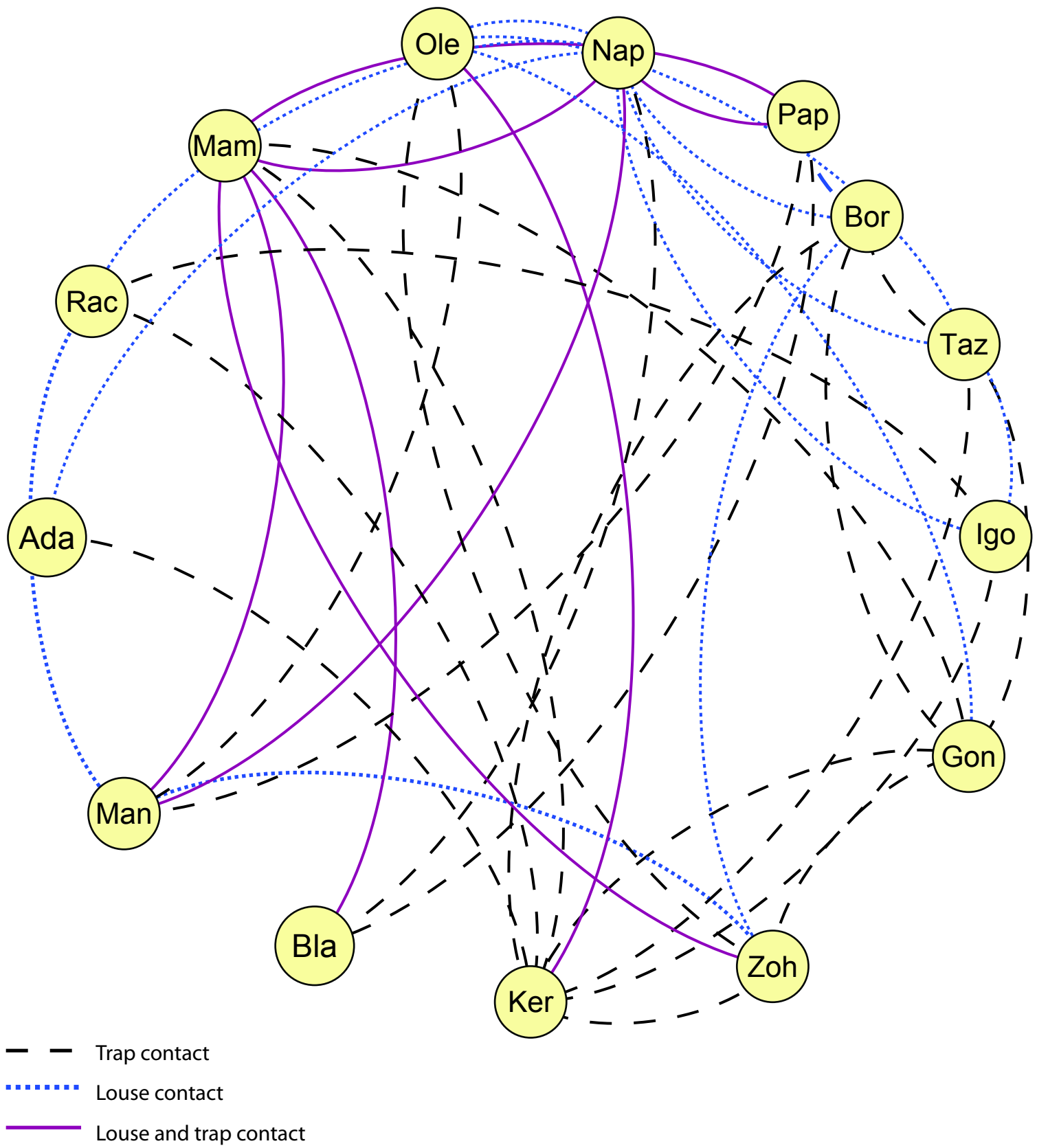

Supplement: Additional file 3 — Figure S3. Social network map of mouse lemurs based on trapping data and louse exchanges. This figure represents the social contacts based on trapping data alone (black dashed line), based on louse transfers (dotted blue line), and contacts that occurred based on both trapping data and louse exchange data (solid purple line). 28 contacts were predicted based on trap locales, and 21 contacts were seen according to louse transfer data. Of the 21 louse exchange contacts, 8 of those were also paired based on trapping data; however, 13 lemur contacts based on louse exchanges can not be explained by trapping data. Of the 8 pairs with overlapping trap and louse contacts, 5 of those pairs belong to one individual (Mam), the same individual found to range widely throughout the trapping transect. These data suggests that while some lemur-lemur contacts may be predicted by trapping data, a majority of the louse exchanges seen in this study could not have been predicted based on trapping data alone. Additionally, the lemurs with the highest eigenvector centrality scores (indicating how well a lemur is connected to other lemurs) differed when calculating networks based on trapping and louse marking data separately. This means that calculating a social network based on trapping data alone would not have exposed the lemur with the most social contacts (Nap) as was revealed using louse marking data. [file 1472-6785-12-4-S3.PDF]

Figure A4.

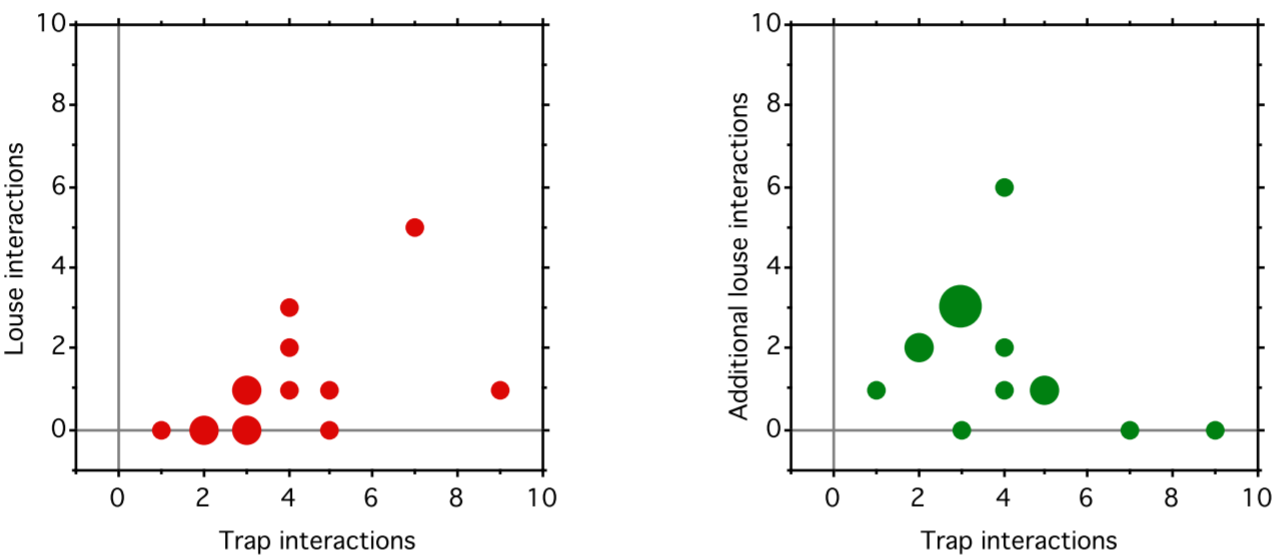

Supplement: Additional file 4 — Figure S4. Individuals with a larger number of social connections calculated using trapping data also have a larger number of these same connections based on louse transfers (left, rs = 0.614, p = 0.027). In addition, louse transfer based calculations reveal additional contacts that could not have been predicted based on trapping data, and hence the number of contacts do not correlate significantly with trapping data based connections (right, rs = -0.417, p = 0.132). The three dot sizes represent one, two, and three individuals. [file 1472-6785-12-4-S4.PDF]

Figure A5.

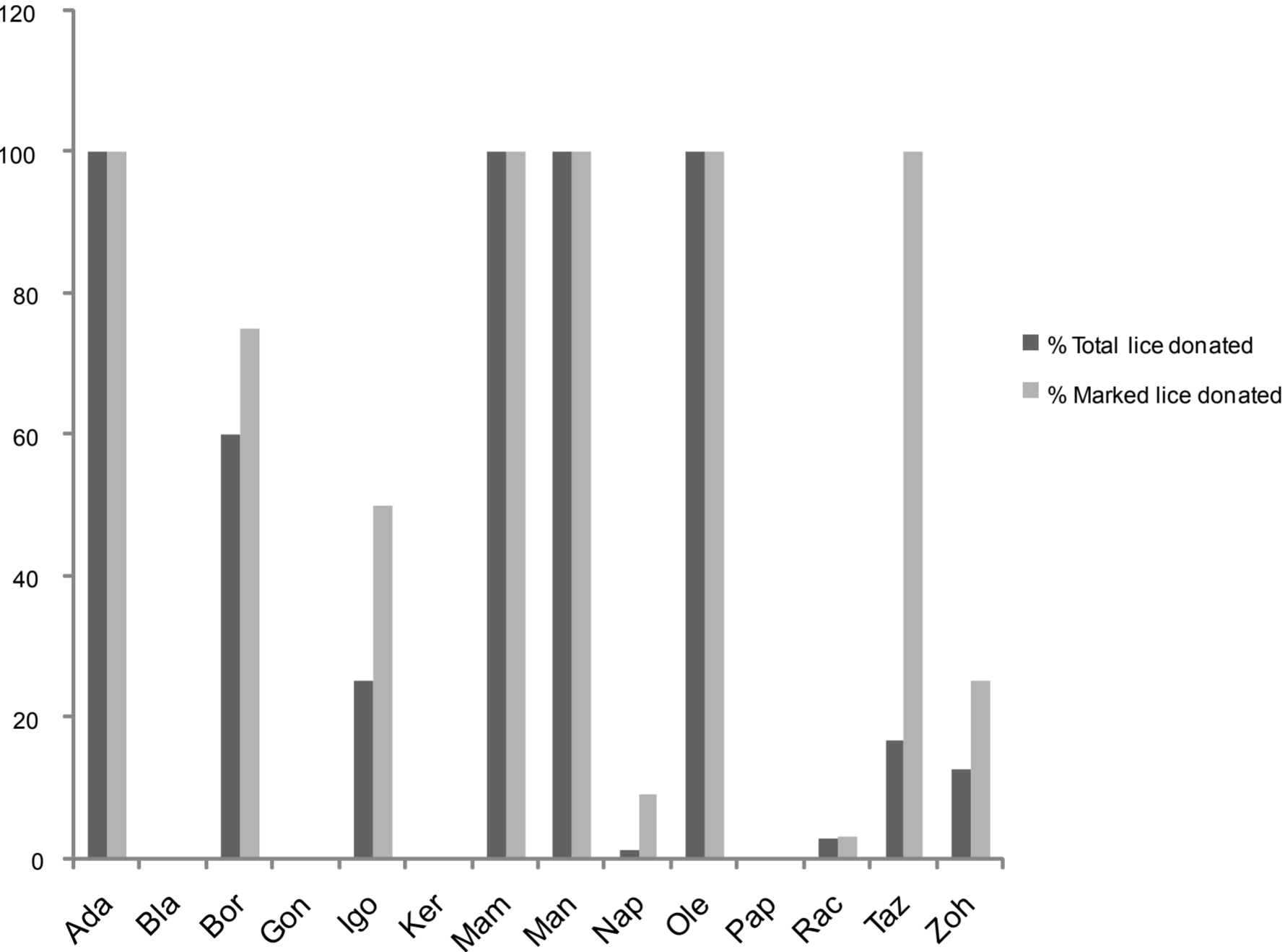

Supplement: Additional file 6 — Figure S5. Histogram of percentage of marked and total lice donated. In this figure the dark grey bars represent the percentage of lice donated out of the total number of lice found on the body. The light grey bars represent the percentage of lice donated out of the total number of marked lice from that individual. When including the total number of lice on the body (both marked and unmarked), five individuals donated more than 50% of their lice, and received on average 0.6 lice. The remaining individuals who donated less than 50% of their total lice received on average 7 lice. This suggests that whether examining the total proportion of lice donated, or the proportion of marked lice that were donated, the trends are the same, and individuals with more lice typically receive more lice from others, and individuals with fewer lice typically donate a larger percentage of them. There is no significant correlation between the total number of lice and the number of lice donated (r = -0.08, p = 0.079), or between the number of lice marked and the number of lice donated (r = 0.036, p = 0.90). [file 1472-6785-12-4-S6.PDF]

Figure A6.

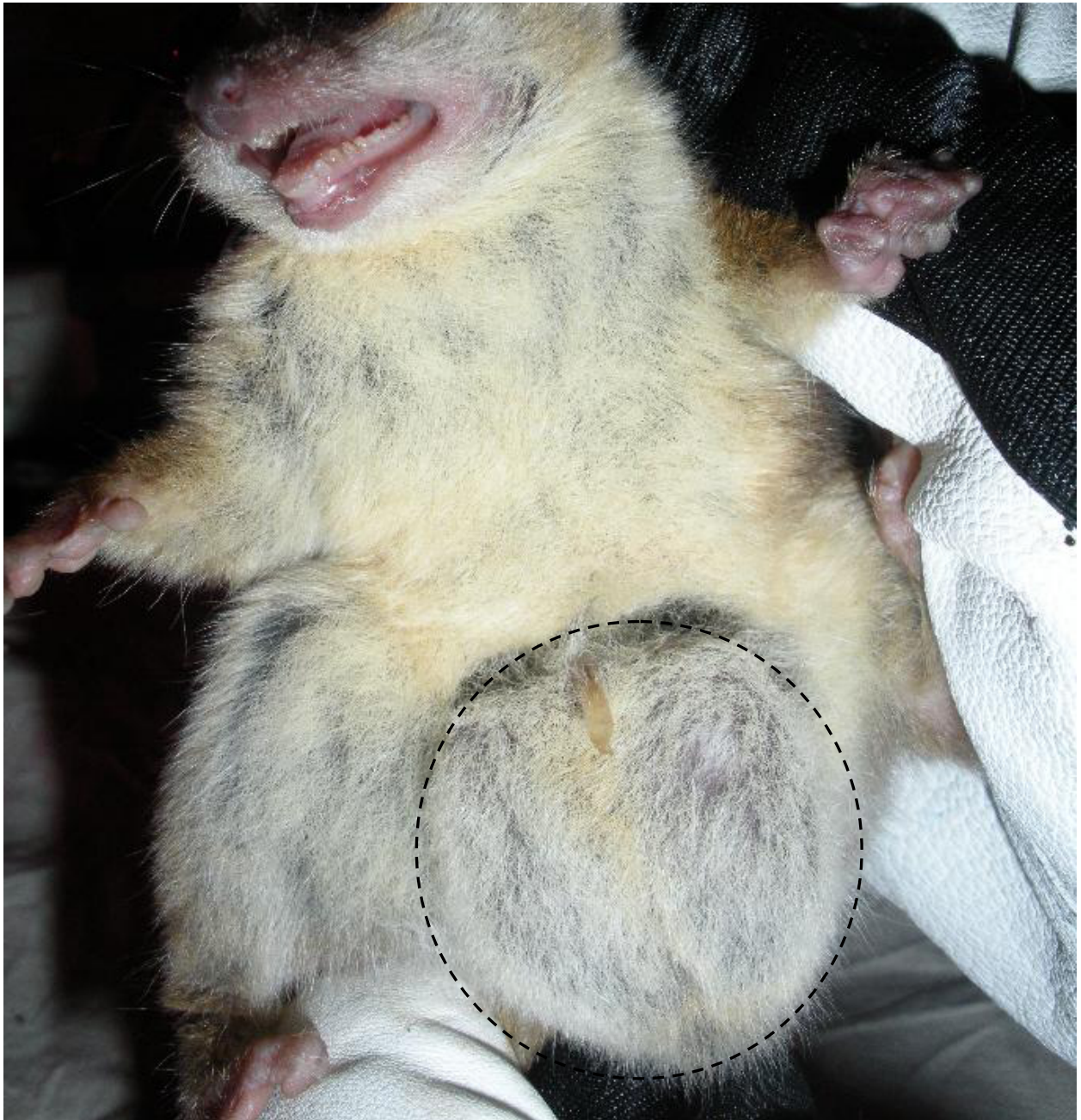

Supplement: Additional file 9 — Figure S6. Testes of a brown mouse lemur during the breeding season. This image demonstrates the seasonal testicular growth seen in male mouse lemurs during the breeding season. The dotted line encircles the testicles. [file 1472-6785-12-4-S9.PDF]
