# Supplementary material for: Mapping the social network: tracking lice in a wild primate (Microcebus rufus) population to infer social contacts and vector potential
Source: BMC Ecol. 2012 Mar 26;12:4. doi: 10.1186/1472-6785-12-4 (PMC3338373; doi:10.1186/1472-6785-12-4)
Supplement: Additional file 5 — Table S1. Table showing eigenvector centralities calculated using social network analysis software. [file 1472-6785-12-4-S5.PDF]

Table A1.

Eigenvector centrality scores

Lemur	Based on louse data	Based on trapping data
Ada	0.24	0.22
Bla	0.16	0.44
Bor	0.47	0.48
Gon	0.10	0.78
Igo	0.35	0.28
Ker	0.10	1.00
Mam	0.66	0.54
Man	0.60	0.97
Nap	1.00	0.53
Ole	0.41	0.48
Pap	0.52	0.53
Rac	0.39	0.28
Taz	0.43	0.48
Zoh	0.42	0.69

Eigenvector centralities calculated using social network analysis program Gephi (Gephi consortium, Paris, France). These results show that according to trapping data Ker is predicted as the lemur with the highest level of connectedness to other lemurs, whereas the louse marking data revealed this to be Nap. The differences here provide further evidence that the social network calculated based on louse marking data could not have been predicted by trapping data alone.
