# Supplementary material for: Mapping the social network: tracking lice in a wild primate (Microcebus rufus) population to infer social contacts and vector potential
Source: BMC Ecol. 2012 Mar 26;12:4. doi: 10.1186/1472-6785-12-4 (PMC3338373; doi:10.1186/1472-6785-12-4)
Supplement: Additional file 7 — Table S2. Table showing the number of donated lice out of the total number of marked lice, and the total number of lice found on the body. [file 1472-6785-12-4-S7.PDF]

Table A2.

Table showing the percentage of donated lice out of the total number of marked and unmarked lice.

Lemur	Total lice	Marked	Donated	Received	% Total lice donated	% Marked lice donated
Ada	5.00	5.00	5.00	0.00	100.00	100.00
Bla	2.00	0.00	0.00	1.00	0.00	0.00
Bor	5.00	4.00	3.00	1.00	60.00	75.00
Gon	6.00	5.00	0.00	1.00	0.00	0.00
Igo	4.00	2.00	1.00	1.00	25.00	50.00
Ker	5.00	2.00	0.00	2.00	0.00	0.00
Mam	9.00	9.00	9.00	0.00	100.00	100.00
Man	5.00	5.00	5.00	2.00	100.00	100.00
Nap	170.00	22.00	2.00	46.00	1.18	9.09
Ole	7.00	7.00	7.00	0.00	100.00	100.00
Pap	12.00	3.00	0.00	5.00	0.00	0.00
Rac	37.00	34.00	1.00	1.00	2.70	2.94
Taz	12.00	2.00	2.00	3.00	16.67	100.00
Zoh	8.00	4.00	1.00	3.00	12.50	25.00

There is no significant correlation between the total number of lice and the number of lice donated ($r= -0.08$, $p=.079$), or between the number of lice marked and the number of lice donated ($r= 0.036$, $p= 0.90$).
