# Supplementary material for: Mapping the social network: tracking lice in a wild primate (Microcebus rufus) population to infer social contacts and vector potential
Source: BMC Ecol. 2012 Mar 26;12:4. doi: 10.1186/1472-6785-12-4 (PMC3338373; doi:10.1186/1472-6785-12-4)
Supplement: Additional file 8 — Table S3. Table representing the maximum distances lemurs travelled according to trapping data along with the maximum distances lemurs travelled according to the movement of their lice. [file 1472-6785-12-4-S8.PDF]

Table A3.

Maximum distances lemurs travelled according to trapping data and louse transfers

Lemur	Trapping data (meters)	Louse transfers (meters)		
Ada	0.00	380.09		
Bla	82.15	64.98		
Bor	92.50	508.80		
Gon	84.20	240.80		
Igo	101.24	580.39		
Ker	403.48	156.82		
Mam	467.30	277.96		
Man	230.88	475.96		
Nap	304.43	633.62		
Ole	33.65	279.29		
Pap	347.33	405.52		
Rac	134.21	633.62		
Taz	92.50	534.03		
Zoh	156.82	277.96		

Maximum distances lemurs moved based on trapping data (left column), and based on louse transfers (right column). There is a significant difference between these distances (p=0.01), with louse transfer distances averaging 390m, and trap-based distances averaging 195 m.
