# Supplementary material for: Mapping the social network: tracking lice in a wild primate (Microcebus rufus) population to infer social contacts and vector potential
Source: BMC Ecol. 2012 Mar 26;12:4. doi: 10.1186/1472-6785-12-4 (PMC3338373; doi:10.1186/1472-6785-12-4)
Supplement: Additional file 10 — Text S1. Additional text. [file 1472-6785-12-4-S10.PDF]

Additional Text S1.

Mouse lemurs are seasonal breeders, and with the onset of the breeding season is a dramatic increase in testicular volume in males. In this population of *Microcebus rufus,* testicular volume peaks in September/October (Fig S6.), and by January most males show a complete regression of testes to a hardly detectable pre-breeding size [12, and this study].

Our method was designed to be instituted without significantly lengthening the amount of time the host is handled and to have minimal influence on the host-parasite relationship and interaction. The following additional data demonstrate that the trapping and handling process had no impact on host parasite intensities and the frequency with which they engaged in louse transfer. Additionally, the materials used to mark lice in this study were tested on adult lice and found to have no deleterious effects, as demonstrated by lack of reaction to application and continued marked-louse survival.

There is no significant correlation between the number of times a host was captured during the study and the mean number of lice observed on them at each capture. Capture rate explains only 2.9% of the variation in mean louse counts.

There is no significant correlation between the number of times a host was captured and the percentage of its marked lice that would be found on other individuals. The number of captures explains only 19% of the variation in values for the percentage of marked lice donated.

There is no correlation between the total number of lice marked on a host and the number of lice that host donates. Total marked louse count explains less than 1% of variation in the number of lice donated. This indicates that lice donated are not simply a product of the number of lice marked.

Though there is a small positive correlation between host age and absolute number of lice transferred ($r= 0.26$), there is no correlation between age and the percentage of marked lice transferred ($r= 0.07$). There is a small positive correlation between testicular volume and number of lice marked ($r= 0.27$).
